# Preparation, Characterization and Biocompatibility of a Silk Fibroin/Bamboo Nanofibrillated Cellulose Composite Hydrogel

**DOI:** 10.3390/gels12010038

**Published:** 2025-12-31

**Authors:** Pan Wu, Chengli Wang, Di Wang, Jiahua Li, Wanfu Yue

**Affiliations:** College of Animal Sciences & Technology, Zhejiang A&F University, Hangzhou 311300, China

**Keywords:** silk fibroin, bamboo nanofibrillated cellulose, composite hydrogel, green material, biocompatibility

## Abstract

To address the limitations of pure silk fibroin (SF) hydrogels, such as poor mechanical strength and rapid degradation, a fully “green” composite hydrogel was developed by integrating bamboo nanofibrillated cellulose (BNC) with SF and crosslinked using the natural agent genipin. The composite formed a stable interpenetrating network, as confirmed by means of SEM and FTIR. This structure led to significantly enhanced mechanical properties (increased storage modulus and pronounced shear-thinning behavior), moderate swelling, and a controllable degradation rate. In vitro biocompatibility assays demonstrated that the BNC-SF hydrogel was non-cytotoxic and excellently supported the adhesion, spreading, and proliferation of L929 fibroblasts. Notably, it exhibited a strong pro-migratory effect in a scratch assay. This work presents a high-performance, injectable scaffold material derived entirely from natural sources, showing great potential for tissue engineering and regenerative medicine applications.

## 1. Introduction

The rapid advancement of tissue engineering imposes increasingly stringent demands on biomaterials. An ideal tissue engineering scaffold must not only possess excellent biocompatibility and controlled degradability but also exhibit suitable mechanical properties and a three-dimensional porous structure to support cell growth and tissue regeneration [1]. Hydrogels play a pivotal role in this field due to their high water content and physical characteristics resembling the natural extracellular matrix [2]. However, many synthetic polymer hydrogels suffer from limitations such as insufficient bioactivity and the potential for degradation products to induce inflammatory responses [3,4]. Consequently, the development of high-performance, “green” hydrogel materials derived from natural sources with tailorable properties has become a major research focus.

Silk fibroin (SF), a natural macromolecular protein extracted from silk, is regarded as a star material for constructing biomedical devices due to its exceptional biocompatibility, tunable biodegradability, low immunogenicity, and remarkable ability to form films and gels [5,6,7]. Furthermore, silk is a renewable and biodegradable green resource, aligning with the principles of sustainable development [8]. Despite these advantages, pure SF hydrogels often exhibit inherent weaknesses, including relatively low mechanical strength, structural brittleness, and an excessively rapid degradation rate in physiological environments, which limit their application as load-bearing or long-term implants [9,10].

To overcome these limitations, compositing SF with other natural polymers or nanofillers presents a highly effective strategy for performance enhancement [11]. Nanocellulose, derived from various plant resources, has emerged as an outstanding green nanomaterial for reinforcing biopolymer matrices. It boasts high specific strength, high modulus, large specific surface area, and excellent biocompatibility [12,13]. Bamboo-derived nanocellulose (BNC) is a nanoscale fibrillar material isolated from the abundant and fast-growing bamboo plant via physical or chemical methods. BNC not only inherits the superb mechanical properties of nanocellulose but is also considered particularly sustainable due to the rapid renewability of its bamboo source [14,15]. Previous studies have demonstrated the effectiveness of nanocellulose (e.g., from wood or bacterial cellulose) in reinforcing SF matrices, primarily improving mechanical properties [16,17]. However, the exploration of BNC, with its unique fibrillar morphology and potential bioactive cues, in creating fully natural composite hydrogels with SF remains less investigated, especially concerning the synergistic control of mechanical, degradation, and pro-regenerative biological functions.

The formation of a stable network is crucial for hydrogel performance. The choice of crosslinking agent directly impacts the material’s mechanical stability, degradation profile, and biocompatibility. Compared to traditional chemical crosslinkers like glutaraldehyde, which raises cytotoxicity concerns [18], genipin—a natural iridoid compound extracted from Gardenia jasminoides fruits—offers superior characteristics. It exhibits significantly lower cytotoxicity and can efficiently react with primary amine groups in proteins (like SF) to form stable, bluish covalent crosslinks, leading to improved mechanical strength and slower degradation [19,20,21]. The use of genipin as a crosslinker greatly enhances the biosafety and green credentials of the fabrication process.

This study aims to develop a high-performance composite hydrogel utilizing a fully natural and renewable “green” material system comprising silk fibroin, bamboo nanocellulose, and genipin. We hypothesize that genipin-mediated crosslinking can facilitate the formation of a synergistically enhanced interpenetrating network between BNC and SF, resulting in a scaffold material with superior comprehensive properties. This work systematically investigates the physicochemical properties and biological performance of this composite material, intending to provide new insights and a solid experimental foundation for developing green, high-performance biomaterials for tissue regeneration and repair.

## 2. Results and Discussion

### 2.1. Microstructure Analysis

**Figure 1:**
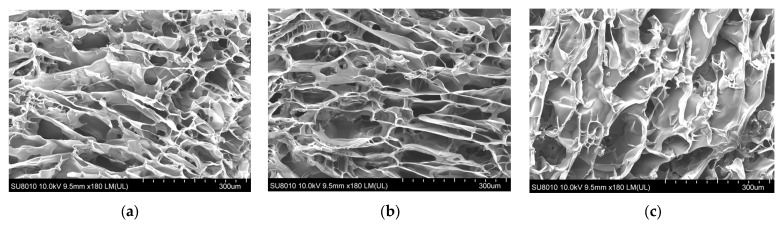
(**a**) The SF-only hydrogel group exhibits a dense lamellar structure formed by the self-assembly of the silk fibroin network, with relatively small but well-interconnected pores.; (**b**) The BNC-only hydrogel group possesses a well-defined, highly ordered fibrillar bundle skeleton with distinct pore boundaries, which can be attributed to the high-strength supporting structure formed by bamboo nanofibrillated cellulose. (**c**) The BNC-SF composite group exhibits a highly cross-linked interpenetrating network structure, characterized by thickened pore walls and distinct three-dimensional support.

The SEM results (Figure 1) clearly reveal the porous architectures of the various hydrogels. The pure SF hydrogel exhibits a characteristic lamellar porous structure formed by the stacking of β-sheets, featuring small but well-interconnected pores, which is associated with the molecular conformation adopted by silk fibroin during its self-assembly process [22]. In contrast, the pure BNC hydrogel displays a regular, fibrous-bundle skeleton constructed from cellulose nanofibrils, with distinct pore boundaries; this rigid framework provides substantial physical support [23]. Remarkably, the BNC-SF composite hydrogel demonstrates a unique interpenetrating polymer network (IPN) structure. Within this network, the silk fibroin lamellae and cellulose nanofibrils are intricately entangled, resulting in significantly thickened pore walls. This observation provides direct morphological evidence that genipin successfully induced crosslinking both between SF molecular chains and at the interface of SF and BNC. The underlying crosslinking mechanism can be elucidated as follows: Genipin, a bifunctional ester cyclic compound, undergoes a nucleophilic attack from the primary amine groups on SF peptide chains. This reaction forms intramolecular and intermolecular covalent crossbridges [10,24]. Concurrently, the abundant surface hydroxyl groups on BNC form an extensive hydrogen-bonding network with polar groups (e.g., -NH_2_, -COOH) on SF [25]. This synergistic combination of covalent crosslinking and physical interactions collaboratively constructs a co-enhanced composite scaffold. The resulting dense and stable microstructure is anticipated to significantly improve the mechanical stability of the hydrogel. Furthermore, it provides an ideal 3D environment conducive to cell growth and the efficient transport of nutrients [26].

### 2.2. Performance Characterization

#### 2.2.1. Chemical Structure Analysis

**Figure 2:**
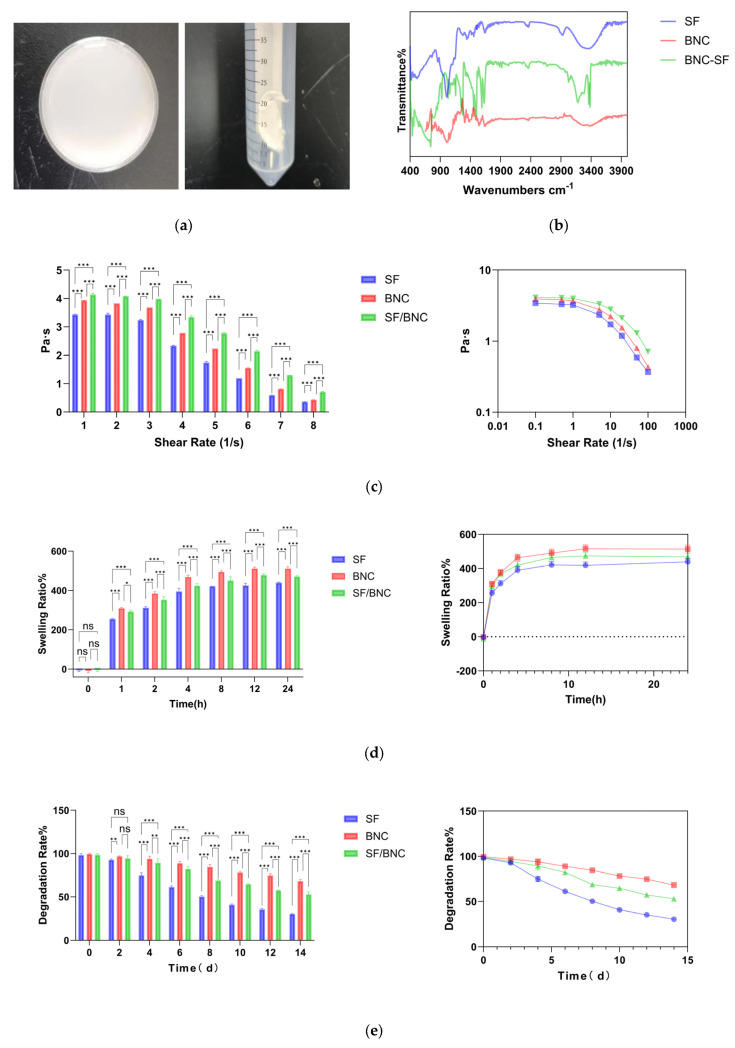
(**a**) Physical image of composite hydrogel; (**b**) Fourier Transform Infrared (FTIR) Spectroscopy Analysis of SF, BNC, and SF/BNC Composite Hydrogels; (**c**) Evolution of Viscosity for SF, BNC, and BNC-SF Hydrogels under Different Shear Rates (0.1–100 s^−1^); (**d**) Swelling Behavior of the Different Hydrogels over 24 h; (**e**) In Vitro Degradation Behavior of the Three Hydrogel Groups in Phosphate-Buffered Saline (PBS, pH 7.4) at 37 °C. (*** indicates *p* < 0.001, ** indicates *p* < 0.01, * indicates *p* < 0.05, and ns indicates no significance. In the figures below, the meaning of * is the same.).

The FTIR spectra (Figure 2b) provide further molecular-level confirmation of the successful composite formation and the interactions therein. The characteristic absorption peaks of SF (Amide I at ~1640 cm^−1^, Amide II at ~1520 cm^−1^) and BNC (C–O–C at ~1050–1030 cm^−1^) are all distinctly present in the spectrum of the composite, indicating successful blending of the two components.Notably, the Amide I band in the composite underwent a slight blueshift (from 1640 cm^−1^ to 1645 cm^−1^), which is typically associated with the involvement of C=O groups in hydrogen bonding and/or a conformational transition of the protein secondary structure towards a β-sheet configuration [27]. Concurrently, the shape of the broad O–H/N–H stretching vibration peak (~3270 cm^−1^) broadened further and its intensity changed. These observations provide strong evidence for the formation of new intermolecular hydrogen bonds between the amino groups of SF and the hydroxyl groups of BNC. Furthermore, they indicate the consumption of primary amine groups due to the cross-linking reaction between genipin and SF, which consequently altered the hydrogen-bonding environment [9,10,14]. These molecular-level interactions form the structural basis for the observed enhancements in the macroscopic properties of the composite hydrogel, such as its improved mechanical strength and slowed degradation rate.

#### 2.2.2. Rheological and Mechanical Properties

The rheological test results (Figure 2c) indicated that all hydrogels exhibited typical gel-like behavior (G′ > G″) and pronounced shear-thinning behavior, which is crucial for minimally invasive implantation via injection [28]. The BNC-SF composite hydrogel demonstrated the highest storage modulus (G′) and complex viscosity across all tested frequencies, confirming the formation of the densest and most stable three-dimensional network structure. This superior mechanical performance is primarily attributed to the following synergistic mechanisms: Firstly, the BNC nanofibers serve as rigid physical crosslinking points and reinforcing fibers, effectively sharing and transferring stress, thereby preventing excessive deformation of the SF network under external force [29]. Secondly, the covalent crosslinked network introduced by genipin provides a stable skeleton for the entire system, significantly enhancing the network’s elasticity. Finally, the extensive hydrogen bonding between SF and BNC acts as reversible physical crosslinks, playing a vital role in energy dissipation [30]. This “rigid-flexible combined” multiple crosslinked network structure represents an effective strategy for achieving high-performance biohydrogels.

#### 2.2.3. Swelling and Degradation Behavior

Swelling experiments (Figure 2d) revealed that the BNC-only hydrogel exhibited the highest equilibrium swelling ratio, which can be attributed to the abundance of hydrophilic hydroxyl groups on its cellulose molecular chains. The swelling ratio of the BNC-SF composite hydrogel was intermediate between those of the pure BNC and SF hydrogels. This indicates that the dense covalent network formed by genipin crosslinking effectively restricts polymer chain relaxation and limits water penetration and absorption [18,31]. A moderate swelling ratio is beneficial for maintaining the dimensional stability of the material after implantation, thereby avoiding compression on surrounding tissues caused by excessive expansion.

The in vitro degradation results (Figure 2e) corresponded well with the swelling behavior. The pure SF hydrogel degraded the most rapidly, primarily due to its reliance on physical crosslinking, which results in poorer network stability and makes the molecular chains more susceptible to dissociation and hydrolysis in PBS [4]. In contrast, the pure BNC hydrogel degraded the slowest, owing to the inherent enzyme resistance and high crystallinity of cellulose [13]. The degradation rate of the BNC-SF composite hydrogel was effectively modulated, falling between the two pure components. This controlled degradability is attributed to two main factors: firstly, the dense interpenetrating network (IPN) structure hinders water penetration and diffusion, thereby slowing down the hydrolysis of SF chain segments. Secondly, the covalent bonds formed by genipin crosslinking exhibit higher stability and are less prone to hydrolytic cleavage [10,32]. This tunable degradation profile allows the material to be matched to the repair cycles of different tissues. For instance, when used as a scaffold for cartilage or skin regeneration, it can provide temporary mechanical support while simultaneously creating space for the ingrowth of new tissue.

### 2.3. Biocompatibility

**Figure 3:**
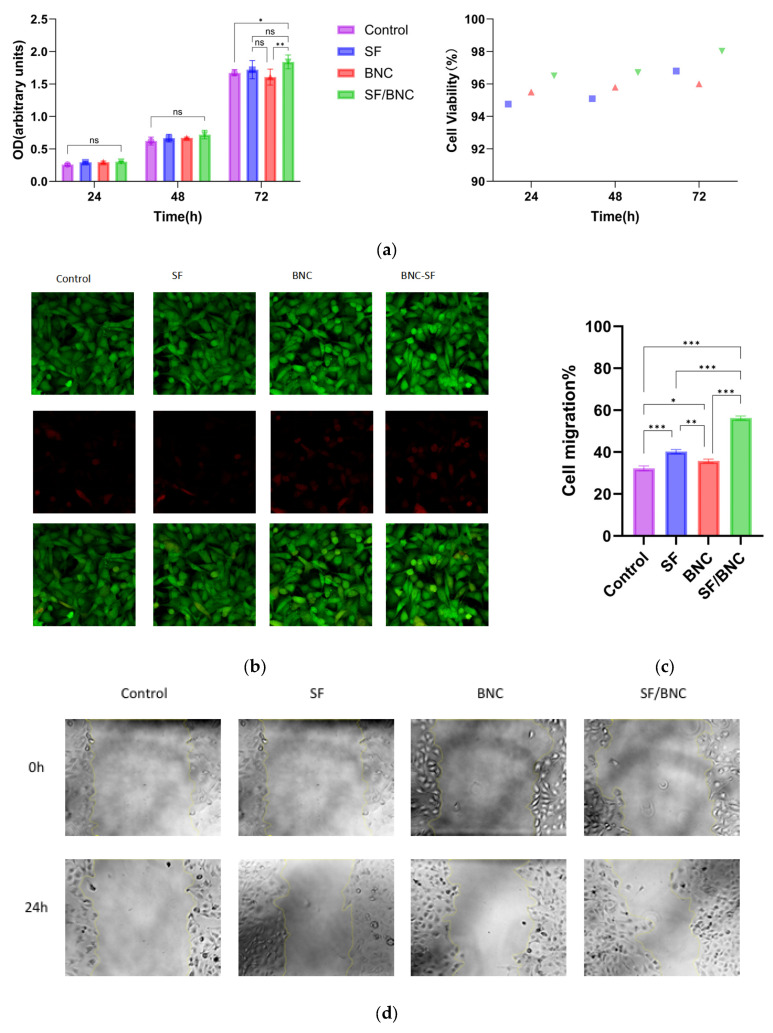
(**a**) Results of the CCK-8 Assay; (**b**) live/dead fluorescence staining images of cells cultured on the Control, SF, BNC, and BNC-SF materials. Viable cells are stained green with Calcein-AM, while dead cells are stained red with propidium iodide (PI); (**c**,**d**) Scratch wound healing assay of cells treated with different materials at 0 h and 24 h. (*** indicates *p* < 0.001, ** indicates *p* < 0.01, * indicates *p* < 0.05, and ns indicates no significance).

To systematically evaluate the biological performance of the prepared composite hydrogel, an in vitro study was conducted using L929 fibroblasts, focusing on three aspects: cytotoxicity, cell adhesion and proliferation capability, and cell migration activity. The CCK-8 assay was used to quantitatively evaluate the effect of material extracts on cell proliferation. As shown in Figure 3a, over a 72-h culture period, the relative proliferation rates of cells in the pure silk fibroin (SF) group, pure bamboo nanocellulose (BNC) group, and composite (BNC-SF) group all remained above 90% compared to the negative control group (culture medium). No statistically significant differences were observed among the groups or between any material group and the control group (*p* > 0.05). These results indicate that the extracts from all three hydrogel materials did not significantly inhibit the proliferative activity of L929 cells, meeting the non-toxic requirements of the ISO 10993-5 [33] standard for cytotoxicity evaluation of medical devices. The composite group showed the highest proliferation rate at 72 h, suggesting that its released microenvironment may be more conducive to long-term cell viability. Cell adhesion, spreading, and viability on the material surfaces were directly observed via live/dead cell staining (Figure 3b). After 48 h of culture, cells in the control group (culture plate) exhibited the typical spindle-shaped, spread morphology of fibroblasts and were evenly distributed. In the material groups, cells adhered well to the hydrogel surfaces. The SF group showed moderate cell density with normal morphology. Cells in the BNC group had a slightly smaller spreading area compared to other groups but maintained good viability. The BNC-SF composite group demonstrated the best cytocompatibility: it not only supported the highest cell density but also exhibited the most typical spreading morphology with well-extended pseudopodia, indicating that the physicochemical properties of the material surface (e.g., hydrophilicity, nanofibrous topography) are more favorable for cell adhesion and early biological behavior [34]. Across all groups, only sporadically distributed dead cells (red fluorescent dots) were observed within the field of view, and their numbers were comparable to those in the control group, further confirming the low cytotoxicity of the materials from a morphological perspective. The scratch wound healing assay (Figure 3c,d) further demonstrated that the extract of the BNC-SF composite hydrogel significantly promoted the migration of L929 fibroblasts. Cell migration is a critical process in tissue regeneration. At 0 h post-scratch, the initial scratch widths were consistent across all groups under the same magnification. After 24 h of culture, both the control group and the groups treated with material extracts showed varying degrees of cell migration. The cell migration rate was 32.2% ± 2.1% for the control group, 40.2% ± 1.1% for the SF group, and 35.7% ± 2.9% for the BNC group. Remarkably, the BNC-SF composite group exhibited the strongest pro-migratory capability, with a cell migration rate as high as 56.3% ± 3.2%. The difference was statistically significant compared to the control and other material groups (*p* < 0.01). Components found in SF degradation products have been shown to possess bioactivity that promotes cell migration [4,35]. Furthermore, BNC nanofibers may release bioactive oligosaccharides during degradation [36], and the stable three-dimensional structure of the composite material provides physical guidance for cell migration. It is plausible that BNC and SF act synergistically during degradation, collectively activating signaling pathways associated with cell migration [27,37]. These results suggest that during degradation or interaction with the microenvironment, the BNC-SF composite hydrogel may release or create biological or physical cues that are more favorable for driving directed cell migration.

In summary, based on the above cellular evaluations, the BNC-SF composite hydrogel exhibits excellent biocompatibility. It is non-cytotoxic, and its stable structure and surface properties effectively promote cell adhesion, spreading, and proliferation. More importantly, the composite hydrogel can significantly accelerate the migration process of fibroblasts. This characteristic is crucial for applications such as cell scaffolds, providing direct experimental evidence for the further application of this material in the field of soft tissue engineering.

## 3. Conclusions

This study successfully developed and systematically evaluated a fully natural-based silk fibroin/bamboo nanofibrillated cellulose (BNC-SF) composite hydrogel. As visually summarized in Table 1, a stable interpenetrating network structure was constructed using the natural crosslinker genipin, which significantly enhanced the overall performance of the material. The main conclusions are as follows: A Green, High-Performance Composite System was Successfully Fabricated: Utilizing silk fibroin and bamboo nanofibrillated cellulose derived from renewable resources, combined with the low-toxicity natural crosslinker genipin, an environmentally friendly strategy for biomaterial preparation was established. FTIR and SEM results confirmed that genipin effectively promoted crosslinking between BNC and SF molecules, forming an interpenetrating network with a dense structure and uniform porosity. Synergistic Enhancement of Physicochemical Properties was Achieved: Compared to single-component hydrogels, the BNC-SF composite hydrogel demonstrated significantly improved mechanical properties (storage modulus and viscosity) and exhibited excellent shear-thinning behavior and injectability. Furthermore, the composite material possessed a moderate swelling ratio and a controllable degradation rate, enabling it to better match the requirements of tissue regeneration. Excellent Biological Functionality was Confirmed: In vitro cell experiments demonstrated that the composite hydrogel is non-cytotoxic and effectively supports cell adhesion and proliferation. Importantly, the scratch assay confirmed its significant ability to promote cell migration. This characteristic renders it particularly advantageous for applications requiring rapid tissue regeneration, such as wound healing. In summary, this study not only verifies the significant synergistic effects between BNC and SF under genipin crosslinking but also highlights the considerable application potential of this composite hydrogel as an injectable scaffold material in the fields of tissue engineering and regenerative medicine. Future work will focus on evaluating its long-term in vivo biocompatibility, degradation behavior, and efficacy in tissue repair.

## 4. Materials and Methods

### 4.1. Materials

Silk fibroin (SF) was extracted from Bombyx mori silk fibers through degumming in an aqueous sodium carbonate solution, followed by dissolution in lithium bromide, dialysis, and lyophilization. The final product was reconstituted as a 6% (*w*/*v*) aqueous solution for subsequent use.

Bamboo nanofibrillated cellulose (BNC) was prepared from three-year-old moso bamboo powder through alkali treatment and bleaching purification, followed by 15 cycles of processing using a high-pressure homogenizer at 120 MPa. The resulting suspension had a solid content of 1.0% (*w*/*v*).

Genipin (purity > 98%) was purchased from Chengle Technology (Hangzhou, China) Co., Ltd. Prior to use, it was dissolved in ultrapure water to prepare a 10 mM stock solution, which was stored protected from light.

The L929 mouse fibroblast cell line was obtained from Qingqi (Shanghai, China) Biotechnology Development Co., Ltd. Cells were cultured in Dulbecco’s Modified Eagle Medium supplemented with 10% fetal bovine serum and 1% penicillin-streptomycin. All cells were maintained in a humidified incubator at 37 °C with 5% CO_2_. Cells used in experiments were within passages 5–15 and confirmed to be free of mycoplasma contamination via PCR detection.

### 4.2. Preparation of Composite Hydrogels

**Figure 4:**
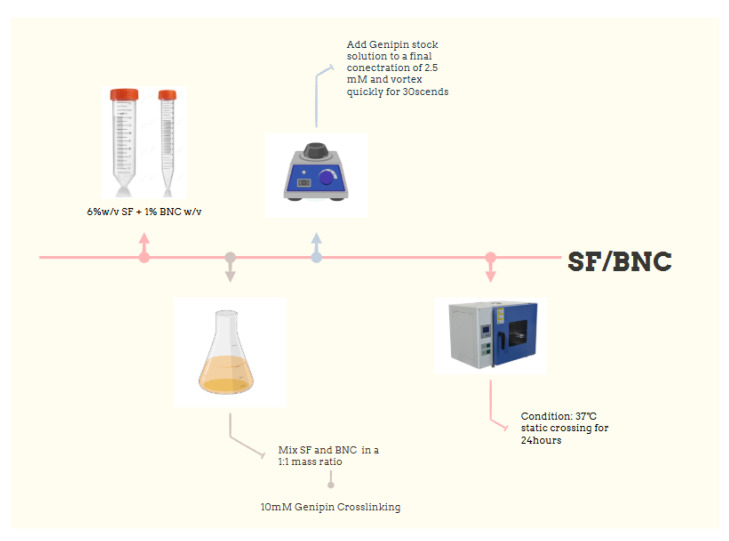
Preparation process of silk fibroin/bamboo nanocellulose composite hydrogel.

The BNC-SF composite hydrogel was prepared using a two-step process involving physical blending followed by chemical crosslinking. As shown in Figure 4, a 6% SF solution and a 1% BNC suspension were blended at a dry mass ratio of 1:1 (SF:BNC) under magnetic stirring at room temperature for 2 h to obtain a homogeneous composite precursor solution. Subsequently, a genipin stock solution was added to this precursor to achieve a final concentration of 2.5 mM in the system. The mixture was quickly vortex-mixed and then transferred into the desired molds, followed by static crosslinking in a 37 °C oven for 24 h to form a stable hydrogel. As controls, pure SF hydrogel and pure BNC hydrogel were prepared under identical conditions by adding the same amount of genipin to SF solution or BNC suspension of the same concentration, respectively.

### 4.3. Material Characterization

The hydrogel samples were rapidly frozen in liquid nitrogen and then fractured. Subsequently, the fractured samples were freeze-dried for 48 h. The dried samples were mounted on sample stubs with the fractured cross-sections facing upward, followed by sputter-coating with a thin layer of gold (approximately 10 nm in thickness) using an ion sputter coater. The internal microstructure and cross-sectional morphology of the hydrogels were observed using a field-emission scanning electron microscope (SEM, model: Hitachi SU8010) at an accelerating voltage of 5.0 kV. The chemical structure and intermolecular interactions of the materials were analyzed using a Fourier transform infrared (FTIR) spectrometer (model: Thermo Scientific Nicolet iS50). Completely dried samples were mixed with spectroscopic-grade potassium bromide at a mass ratio of approximately 1:100, ground thoroughly, and pressed into transparent pellets. Spectra were collected over a wavenumber range of 500–4000 cm^−1^ with a resolution of 4 cm^−1^ and an accumulation of 32 scans. The baseline was corrected using air as the background. The viscoelastic properties of the hydrogels were evaluated using a rotational rheometer (model: TA Instruments DHR-2) equipped with a 20 mm diameter parallel-plate geometry. All tests were conducted at a constant temperature of 25 °C. For frequency sweep tests, measurements were performed within the linear viscoelastic region at a strain of 1%, over a frequency range of 0.1–10 Hz, to record the storage modulus (G′) and loss modulus (G″). For steady-state shear tests, the apparent viscosity of the samples was measured over a shear rate range of 0.1–100 s^−1^ to assess their shear-thinning behavior. All tests were performed in triplicate (*n* = 3). Precisely weighed, fully dried hydrogel samples (initial dry weight M_0_) were immersed in an excess of phosphate-buffered saline (PBS) and allowed to swell in a constant-temperature water bath at 37 °C. At predetermined time points (1, 2, 4, 8, and 24 h), the samples were removed from the PBS. After gently blotting the surface with filter paper to remove excess moisture, the wet weight (M_t) was immediately recorded. The swelling ratio (SR) was calculated according to Equation (1). Each sample group was tested in triplicate (*n* = 3), and the results are expressed as mean ± standard deviation.

(1) $$\mathrm{Swelling}\;\mathrm{Ratio}=\frac{M_{t}-M_{0}}{M_{0}}\times 100\%$$

The initial mass of the dried hydrogel samples was accurately weighed (W_0_), after which they were immersed in PBS containing 0.02% (*w*/*v*) NaN_3_ (as a bacteriostatic agent) and incubated at 37 °C in a constant-temperature shaker with oscillatory agitation at 60 rpm. The PBS solution was refreshed every 48 h. At predetermined time points (days 1, 3, 7, 14, and 21), the samples were removed, rinsed with deionized water, freeze-dried, and weighed again to obtain the dry mass (W_t_). The percentage of remaining mass and the degradation rate were calculated according to Equation (2). Each group was tested in triplicate (*n* = 3).

(2) $$\mathrm{Degradation}\;(100\%)=1-\frac{W_{t}}{W_{0}}\times 100\%$$

### 4.4. Cell Experiment

The extract test was conducted in accordance with the ISO 10993-5 standard. Sterile hydrogel samples were immersed in complete culture medium at a ratio of 1 cm^2^/mL and extracted for 24 h at 37 °C under 5% CO_2_ to obtain the stock extract solution. L929 cells were seeded into 96-well plates at a density of 5 × 10^3^ cells per well and cultured for 24 h to allow attachment. The original medium was then aspirated and replaced with 100 µL of either different concentrations of extract (stock solution, 50% dilution) or fresh complete medium (serving as the negative control). After further incubation for 24, 48, and 72 h, respectively, 10 µL of CCK-8 reagent was added to each well, followed by an additional 2-h incubation. The absorbance at 450 nm was measured using a microplate reader. The relative cell proliferation rate was calculated with the negative control group set as 100%. The experiment was independently repeated three times with six replicates each time (biological replicates n = 3, technical replicates n = 6). Intergroup comparisons were performed using one-way ANOVA, with Tukey’s test for post-hoc analysis, and the significance level was set at *p* < 0.05. Sterilized circular hydrogel discs were placed at the bottom of 24-well plates. L929 cells were seeded directly onto the surface of the hydrogels at a density of 2 × 10^4^ cells per well and cultured for 48 h. Subsequently, the culture medium was aspirated, and the samples were rinsed once with PBS. A working solution containing 2 μM Calcein-AM and 4 μM propidium iodide was added, and the samples were incubated at 37 °C in the dark for 20 min for staining. After staining, the samples were gently rinsed with PBS and immediately observed and photographed under a fluorescence inverted microscope. Viable cells were labeled with green fluorescence by Calcein-AM, while dead cells were labeled with red fluorescence by propidium iodide. The experiment was independently repeated three times.

L929 cells were seeded at a high density of 5 × 10^5^ cells per well in 24-well plates and cultured until a 100% confluent monolayer was formed. A straight scratch was created in the center of the cell monolayer in each well using a sterile 200 µL pipette tip. The wells were gently washed twice with PBS to remove detached cells. Subsequently, the experimental groups were replenished with fresh medium containing 50% hydrogel extract, while the control group received fresh complete medium. Images of the scratch were captured at the same location at 0 h and 24 h post-scratching using an inverted microscope with a 10× objective. The scratch area was measured using Image J1.54 software, and the migration rate was calculated according to Equation (1). The experiment was independently repeated three times, with at least three random fields analyzed per replicate.

(3) $$\mathrm{Migration}\;\mathrm{Rate}=\frac{{Area}_{0h}-{Area}_{24h}}{{Area}_{0h}}\times 100\%$$

### 4.5. Statistical Analysis

All quantitative experimental data are presented as the mean ± standard deviation. Statistical analysis was performed using GraphPad Prism software (version 10.1.2). For comparisons among multiple groups, one-way analysis of variance (ANOVA) was employed. If the assumption of homogeneity of variances was met, Tukey’s multiple comparisons test was applied; if not, Dunnett’s T3 test was used. A *p*-value of less than 0.05 was considered statistically significant.

## Figures and Tables

**Table 1:** Comprehensive comparison of key properties among BNC-SF composite hydrogel and single-component hydrogels.

Performance Indicator	Pure SF Hydrogel	Pure BNC Hydrogel	BNC-SF Composite Hydrogel	Comprehensive Evaluation
**Mechanical Properties**	Low	Medium	High	Significantly enhanced
**Structural Stability**	Layered porous	Fibrous bundle skeleton	IPN interpenetrating network	Most stable
**Degradation Rate**	Fast	Slow	Controllable	Adjustable
**Cytocompatibility**	Good	Good	Excellent	Optimal
**Cell Migration Promotion**	Present	Present	Significant	Strongest
**Injectability**	Good	Poor	Excellent	Most suitable for injection
**Green Attributes**	High	High	High	Fully natural components

## Data Availability

The original contributions presented in this study are included in the article. Further inquiries can be directed to the corresponding author.

## Abbreviations

The following abbreviations are used in this manuscript:

SF	Silk Fibroin
BNC	Bamboo Nanofibrillated Cellulose
PBS	Phosphate-Buffered Saline
SEM	Scanning Electron Microscopy
IPN	Interpenetrating Polymer Network
CCK-8	Cell Counting Kit-8
PI	Propidium Iodide
ISO	International Organization for Standardization
L929	Mouse connective tissue fibroblast cell line
